# Single-Copy Knock-In Loci for Defined Gene Expression in *Caenorhabditis elegans*

**DOI:** 10.1534/g3.119.400314

**Published:** 2019-05-07

**Authors:** Carlos G. Silva-García, Anne Lanjuin, Caroline Heintz, Sneha Dutta, Nicole M. Clark, William B. Mair

**Affiliations:** Department of Genetics and Complex Diseases, Harvard T. H. Chan School of Public Health, Harvard University, Boston, Massachusetts 02115

**Keywords:** *C. elegans*, methods, CRISPR

## Abstract

We have generated a single-copy knock-in loci for defined gene expression (SKI LODGE) system to insert any DNA by CRISPR/Cas9 at defined safe harbors in the *Caenorhabditis elegans* genome. Utilizing a single crRNA guide, which also acts as a Co-CRISPR enrichment marker, any DNA sequence can be introduced as a single copy, regulated by different tissue-specific promoters. The SKI LODGE system provides a fast, economical, and effective approach for generating single-copy ectopic transgenes in *C. elegans*.

The *C. elegans* community has developed multiple protocols to express transgenes in this genetic model. These protocols include extrachromosomal arrays (Mello *et al.* 1991), gamma/UV integration (Evans 2006), biolistic transformation (Praitis *et al.* 2001), and Mos1-mediated single copy insertion (MosSCI) (Frøkjær-Jensen *et al.* 2008). The use of these tools has expedited our understanding of innumerable molecular and physiological mechanisms. However, many issues remain with these systems that limit efficacy, including inter-individual variability in expression levels, potential for disruption of one or more endogenous genes, laborious methodologies, and co-selection/rescue markers (*i.e.*, unc or roller) linked to the transgene that can influence *C. elegans* physiology. To circumnavigate these issues, we have taken advantage of precise and rapid CRISPR/Cas9 genome editing (Dickinson *et al.* 2013; Friedland *et al.* 2013) to make a **s**ingle-copy **k**nock-**i**n **lo**ci for **d**efined **g**ene **e**xpression (SKI LODGE) system at safe harbors in the *C. elegans* genome. The SKI LODGE system allows rapid single copy tissue-specific expression of any gene. SKI LODGE uses simple PCR amplicons as repair templates along with a single well characterized targeting sequence (*dpy-10* crRNA sequence), that also facilitates Co-CRISPR selection to enrich for mutants. Furthermore, after outcrossing, SKI LODGE does not leave other alterations in the genome that may be detrimental to the organism (*e.g.*, rescue sequences used for selection, or random insertional events that can disrupt untargeted coding or regulatory sequences), and can facilitate rapid generation of stably expressed, tissue-specific transgenes.

## Materials and Methods

**Figure 1:**
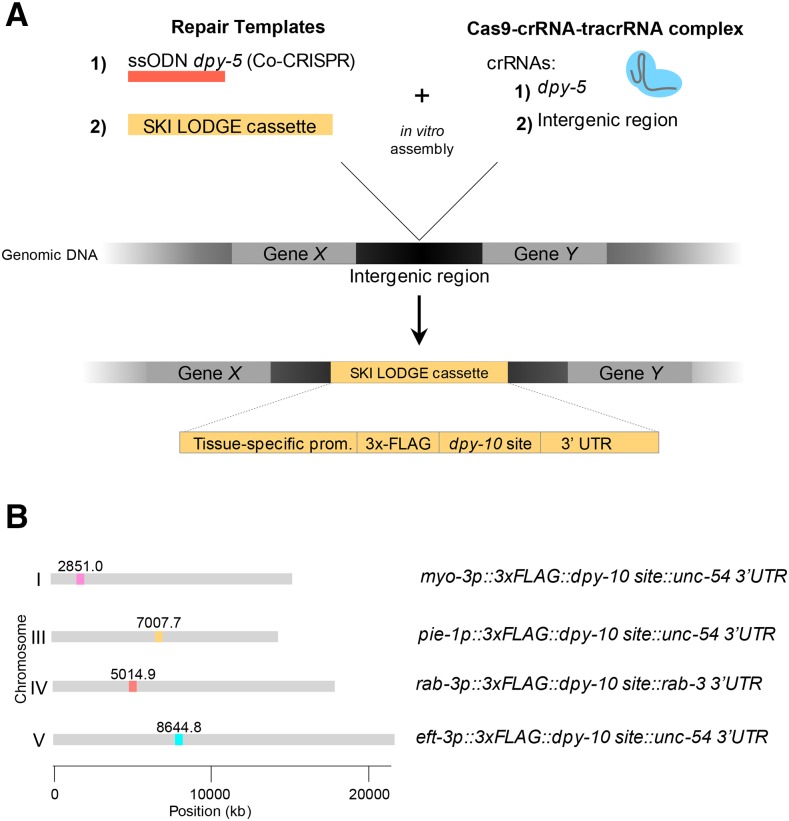
Generation of SKI LODGE lines. (A) Schematic of the SKI LODGE cassettes. Every SKI LODGE cassette was introduced into a defined chromosomal location. SKI LODGE PCR template(s) was combined with the CRISPR/Cas9 complex *in vitro*. This reaction mix was then injected into wild type animals. (Details about SKI LODGE construction can be accessed in Methods section). (B) Genomic locations of SKI LODGE insertions and composition of each cassette.

**Figure 2:**
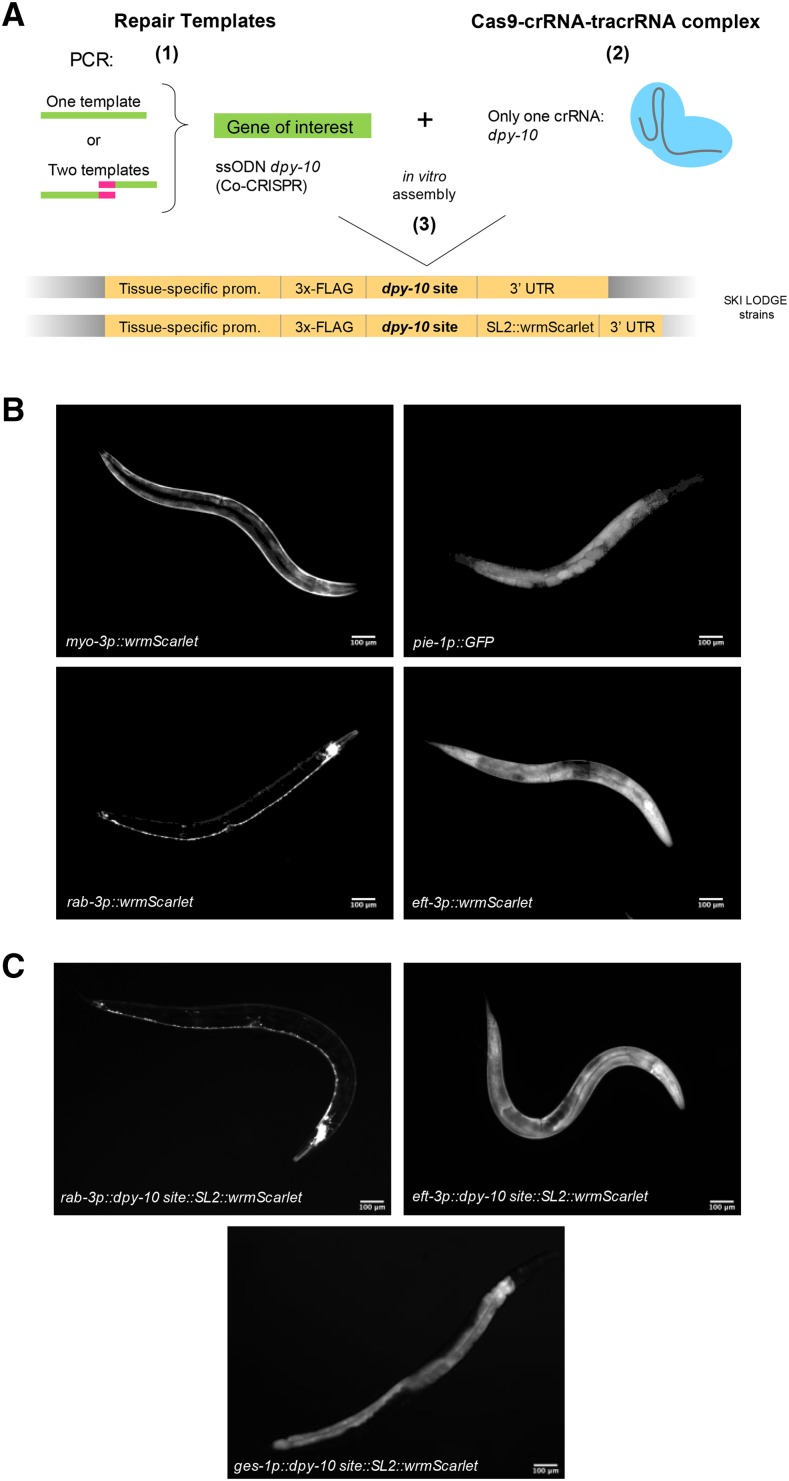
Using the SKI LODGE system to generate single-copy insertions. (A) Flow chart for the generation of single-copy insertions using the SKI LODGE system. (1) Select the SKI LODGE strain to express your gene of interest. Design primers to amplify your gene as homology repair template, including ∼35 bp stretches of SKI LODGE sequences immediately 5′ and 3′ to the *dpy-10* site. (2) Assemble CRISPR/Cas9 complex *in vitro*. (3) Inject pre-assembled CRISPR mix into desired SKI LODGE strain. 3-4 days post injection, isolate individual dumpy/rollers animals, and screen for desired insertion. See *step by step* guide in Methods section for details. (B) Each SKI LODGE strain was tested by knock-in of a fluorescent protein to confirm tissue-specific expression. wrmScarlet was used to confirm ubiquitous, muscle and neuronal expression from the *eft-3*, *myo-3*, and *rab-3* SKI LODGE cassettes, respectively. GFP was used to confirm germline expression in the *pie-1* SKI LODGE. (c) *dpy-10 crRNA*::*SL2* sequence was introduced into *eft-3p*::*wrmScarlet* and *rab-3p*::*wrmScarlet* strains to generate alternative SKI LODGE lines. Animals pictured are at day 1 of adulthood.

We sought to generate transgenic *C. elegans* strains in which a single copy tissue-specific promoter had been knocked in at a defined safe harbor locus, along with the target sequence of a well characterized crRNA that could later be used to knock in any DNA of choice by CRISPR/Cas9 (Figure 1A and Table 1). Since the discovery of genome editing via CRISPR/Cas9, many methods have been developed to make genomic edits in *C. elegans*. One of these methods includes entirely cloning-free steps and direct injection of *in vitro*-assembled Cas9-CRISPR RNA (crRNA) *trans*-activating crRNA (tracrRNA) ribonucleoprotein complexes into the *C. elegans* gonad (Paix *et al.* 2015, 2016). Utilizing this protocol as a base, we developed a toolkit to generate single copy insertions using only one crRNA guide. To define safe harbor loci for the SKI LODGE system, we used those that are well characterized by the MosSCI community (Frøkjær-Jensen *et al.* 2014) and are known to give stable expression with no silencing (Figure 1B). We generated a suite of transgenic cassettes that have a common general design. Each SKI LODGE consists of a tissue-specific promoter, followed by an epitope tag, a highly efficient CRISPR target sequence copied from the *dpy-10* gene, and a 3′ UTR for stable expression: tissue-specific promoter::3xFLAG::*dpy-10* protospacer & PAM::3′UTR (Figure 1). By inserting 30 bases of protospacer and PAM sequence from *dpy-10* gene (Arribere *et al.* 2014), hereafter referred to as “*dpy-10* site” we can simultaneously induce double-stranded breaks at both the transplanted SKI LODGE *dpy-10* site and the endogenous *dpy-10* locus using a single crRNA guide (El Mouridi *et al.* 2017) (Figure 2A). In addition, due to the high efficiency of the *dpy-10* site, the likelihood of getting a template inserted into the SKI LODGE cassette is amplified. In order to introduce the *dpy-10* site into the SKI LODGE cassettes, we established another easily identifiable Co-CRISPR target gene, *dpy-5* (Figure 1A). Finally, we also added an N-terminal 3xFLAG (Figure 1A), which can be used for cell type specific biochemical applications.

**Table 1:** SKI LODGE strains

Strain	Short description	Expression	Chromosome	Verified Expression With:
WBM1119	*pie-1* promoter	Germline	III	GFP
WBM1126	*myo-3* promoter	Muscles	I	wrmScarlet
WBM1140	*eft-3* promoter	Ubiquitous	V	wrmScarlet
WBM1141	*rab-3* promoter	Neurons	IV	wrmScarlet
WBM1179	*eft-3* promoter	Ubiquitous	IV	GFP
WBM1214	*eft-3* promoter + SL2::wrmScarlet	Ubiquitous	V	N/A
WBM1215	*rab-3* promoter + SL2::wrmScarlet	Neurons	IV	N/A
WBM1216	*ges-1* promoter + SL2::wrmScarlet	Intestine	V	N/A

Initially we generated four SKI LODGE strains with differing spatial expression (Table 1), with each SKI LODGE cassette introduced in an intergenic region and on different chromosomes (Figure 1B). Harboring the cassettes on different chromosomes allows the user to cross different SKI LODGE lines into each other. The SKI LODGE lines initially generated include ubiquitous (*eft-3p -eef-1A.1p*-), neuronal (*rab-3p*), muscle (*myo-3p*), and germline (*pie-1p*) promoters. We generated all strains following the Paix *et al.* protocol (Paix *et al.* 2015, 2016) (See File S1 Supplemental Materials and Methods, and Figures 1A,B and S1). After each edit, strains were outcrossed to remove any off-target and Co-CRISPR marker mutations. We modified the method of SKI LODGE cassette insertion into the *C. elegans* genome depending on the size of each promoter sequence. We did one (*rab-3*, *eft-3* and *pie-1*) or two (*myo-3*) edit steps to generate the final cassettes (Figure S1). *rab-3*, *eft-3* and *pie-1* were inserted using two overlapping PCR fragments, and one template was used for *myo-3*, (Figure S1). All final SKI LODGE lines were outcrossed six times, and subsequently assayed for fertility, embryonic lethality, developmental timing and lifespan (Figure S2). Across all parameters tested, SKI LODGE strains were indistinguishable from wild type. In addition, we also checked potential off-target mutations that might result from use of the *dpy-5* Co-CRISPR target gene. We sequenced the three closest genes to each SKI LODGE site that had potential for off target mutation (See File S1). After sequencing, we did not find mutations, and in concordance with the phenotypic assays (Figure S2), these data suggest that SKI LODGE lines have a wild type phenotype, which will markedly facilitate microinjections.

## Data availability

Supplemental Materials and Methods can be found in File S1. Strains and plasmids are available upon request. The authors affirm that all data necessary for confirming the conclusions of the article are present within the article, figures, and tables. Supplemental material available at FigShare: https://doi.org/10.25387/g3.8085740.

## Results

To verify that our SKI LODGE strains could indeed be used to drive tissue-specific gene expression, we first tested all of them by CRISPR knock-in of the wrmScarlet fluorescent protein (El Mouridi *et al.* 2017). We amplified wrmScarlet with ∼35 bases homology arms for 3xFLAG (*myo-3*, *pie-1*, *rab-3* and *eft-3* cassettes) in the left side, and ∼35 bases homology arms for *unc-54 3′UTR* (*myo-3*, *pie-1* and *eft-3* cassettes) or *rab-3 3′UTR* (*rab-3* cassette) in the right side. CRISPR/Cas9 mix was assembled *in vitro* (Paix *et al.* 2015) using purified Cas9 protein. As predicted, utilizing only one crRNA guide (Figure 2A), we were able to obtain *dpy-10* mutant animals that also contained inserted wrmScarlet into the SKI LODGE cassette. We observed expected patterns of tissue-specific expression for wrmScarlet driven by *eft-3*, *myo-3* and *rab-3* promoters (Figures 2B and S3). However, we did not observe expression of wrmScarlet in the germline of the *pie-1* strain (data not shown). wrmScarlet does not have introns (El Mouridi *et al.* 2017), which greatly enhance transgene expression (Boulin *et al.* 2006). To test whether lack of intronic sequence in wrmScarlet might have impacted germline expression, we inserted GFP with artificial introns into the *pie-1* SKI LODGE. Using GFP (with intronic sequences) as a template, the *pie-1* SKI LODGE cassette drove GFP expression in the germline (Figures 2B and S3). We also observed wrmScarlet intestinal ectopic expression in our initial SKI LODGE single-copy *rab-3p* (neuronal) strain that contained the *unc-54 3′UTR* (data not shown). Since, 3′UTRs can modulate gene expression in *C. elegans* (Merritt *et al.* 2008), and *unc-54* lies upstream of *aex-5* which is expressed in intestine, we generated an additional *rab-3p* SKI LODGE line, swapping out the *unc-54 3′UTR* for the *rab-3 3′UTR*. Knock-in of wrmScarlet into the *rab-3p* with the *rab-3* 3′UTR SKI LODGE line resulted in no identifiable expression in the intestine at day one and six of adulthood (Figures 2B and S3), suggesting this line can be used to more cleanly drive gene expression in the *C. elegans* nervous system, and that use of the *unc-54* 3′UTR rather than high copy number may explain previous examples of non-neuronal leaky expression for pan-neuronal promoters such as *rab-3* (Wang *et al.* 2016). All tested SKI LODGE lines were outcrossed at least four times and verified for off-target events of *dpy-10*, particularly in the R12E2.15 gene. After sequencing, we did not find mutations in this region (See File S1). Lastly, we generated three SKI LODGE lines that introduce *gpd-2* SL2 *trans*-splicing sequence between 3xFLAG and wrmScarlet: *eft-3p*::*3xFLAG*::*wrmScarlet*, *rab-3p*::*3xFLAG*::*wrmScarlet*, and *ges-1p*::*3xFLAG*::*wrmScarlet* (Figure 2C and File S1). Using these lines, a gene of interest can be co-expressed with wrmScarlet without generating a gene::wrmScarlet translational fusion reporter.

We have successfully introduced templates of different sizes into different SKI LODGE lines (Table S1). The shortest template introduced was 774 bp (wrmScarlet) with a frequency of up to 13.04% of F1 animals. As the template increased in size, the frequency decreased: 0.44% of 1872 bp, and 2.08% of 2319 bp. We also introduced two templates at the same time with overlapping sequences with a frequency of 13.39% of 1534 bp and 778 bp; and 9.56% of 829 bp and 778 bp. All these insertions were made following Paix protocol (Paix *et al.* 2015, 2016) using PCR products with blunt-strand ends and with at least 35 bp of homology arms. Recently, Dokshin *et al.* (Dokshin *et al.* 2018) proposed that PCR products with single-strand ends work more efficiently than products with blunt-strand ends. Single-strand ended PCR products are hybrid asymmetric molecules originated from two donors: one with 120 bp homology arms and the other with no homology arms (Dokshin *et al.* 2018). To compare blunt- *vs.* single-strand ends in some of our SKI LODGE lines, we introduced wrmScarlet with single-strand ends (See File S1). We observed similar frequency of insertion in the SKI LODGE *myo-3* strain in both blunt- and single-strand ends, 13.04% and 13.33%, respectively (Table S1). The frequency of insertion increased when we introduced wrmScarlet into the SKI LODGE *eft-3* strain, from 8.57% (blunt-strand ends) to 26% (single-strand ends) (Table S1). Overall, both protocols, Paix and Dokshin, can be used in the SKI LODGE lines, but the efficiency of insertion will depend on several factors. For example, in our hands, we have noted that a successful CRISPR edit relies on template length, complexity of template sequence, template concentration, size of homology arms, and microinjection proficiency. Thus, while short sequences (*i.e.*, 3xFLAG tag) are easily inserted, repetitive sequences (*i.e.*, those located in promoters) reduce efficiency. Large homology arms increase frequency of insertion, and a well-honed microinjection technique is crucial to obtain the CRISPR edit.

## Discussion

In summary, the SKI LODGE system allows insertion of single-copy transgenes in *C. elegans* into safe harbor loci using CRISPR/Cas9 editing in one week (Figure 2A and Table 1). This protocol has several advantages over existing methods. These include, the ease of CRISPR knock-in using a *dpy-10* crRNA guide both for knock-in and Co-CRISPR edits, and reduced time and cost due to the use of PCR amplicons and a single crRNA guide. SKI LODGE strains are phenotypically wild type, and as such are easier to inject into than mutant strains, such as *unc-119* animals often used in other methods (Praitis *et al.* 2001; Frøkjær-Jensen *et al.* 2014). The final generated transgenes are single copy, expressed at known loci that do not impact endogenous gene expression, and do not contain additional material such as selection markers or rescue constructs that impact their utility. SKI LODGE also facilitates optional tissue-specific epitope tagging for future biochemical applications such as IP, ChIP, IF, or Western blotting. SKI LODGE lines can be used to insert one or two templates (with overlapping sequences) at the same time (Figure 2A). However, for large insertions (>3000 bp) we recommend following protocols that use plasmid templates with long homology arms (Dickinson and Goldstein 2016). We will continue to develop new SKI LODGE lines with enhanced application, and encourage the community to do the same (see Table S2 for pipeline). All SKI LODGE lines are available freely to the *C. elegans* community, and a step by step user guide for use can be found in the File S2. Finally, strains reported here, new strains, updated protocols, and all sequences can be found at www.themairlab.com/skilodge.
